# Serological and biochemical characterization of Aspergillus fumigatus Asp f 10 as a potential diagnostic marker in ABPA and related respiratory diseases

**DOI:** 10.3389/falgy.2026.1733299

**Published:** 2026-06-05

**Authors:** Shramana Ghosh, Angira Dasgupta, Rajat Kanti Sarkar, Chandrabati Chatterjee, Mahesh Padukudru Anand, Kashinath Bhattacharya, Utpal Bakshi, Gaurab Sircar

**Affiliations:** 1 Department of Botany, Visva-Bharati, Santiniketan, West Bengal, India; 2 Department of Chest Medicines, B. R. Singh Hospital and Centre for Medical Education and Research, Kolkata, West Bengal, India; 3 Institute of Health Sciences, Presidency University, Kolkata, West Bengal, India; 4 Department of Respiratory Medicine, JSS Medical College, JSSAHER, Mysore, Karnataka, India

**Keywords:** ABPA, Asp f 10, asperg illus, diagnostic marker, IgE, IgG

## Abstract

**Background:**

*Aspergillus fumigatus* is a major cause of allergic bronchopulmonary aspergillosis (ABPA), yet diagnosis remains challenging due to overlap with other atopic conditions. Asp f 10, a secreted aspartic protease, is listed as an allergen but remains poorly characterized in terms of biochemical properties and diagnostic relevance.

**Methods:**

We profiled the *A. fumigatus* secretome under host-mimicking conditions to identify IgG-reactive proteins, followed by cloning and recombinant expression of full-length and mature Asp f 10. Biochemical stability, enzymatic activity, and serological reactivity (IgE and IgG) were evaluated using sera from patients with ABPA, steroid-unresponsive severe asthma with *Aspergillus* sensitization (SAAS), and other allergic and non-allergic controls. Diagnostic performance was assessed by ROC and linear discriminant analyses.

**Results:**

Asp f 10 was identified as an extracellular IgG-reactive protease. Recombinant mature Asp f 10 exhibited folded conformation, structural stability, and gelatinolytic activity, whereas the full-length protein lacked defined structure. Both isoforms bound patient IgE and IgG; however, only Asp f 10–specific IgG responses were significantly elevated in ABPA and SAAS. ROC analysis demonstrated excellent diagnostic capacity for IgG (AUC = 0.90), far surpassing IgE (AUC = 0.54). Despite IgE recognition, mature Asp f 10 failed to induce histamine release, suggesting limited effector activity.

**Conclusion:**

Asp f 10 is a biologically active protease, and Asp f 10–specific IgG responses provide a robust biomarker for ABPA, outperforming IgE in diagnostic accuracy. Incorporating Asp f 10 into serological panels may improve differential diagnosis of *Aspergillus*-associated respiratory diseases.

## Introduction

*Aspergillus fumigatus* (AF) is a ubiquitous saprophytic mold and one of the most clinically significant fungal pathogens, particularly in the context of respiratory health. This opportunistic fungus is frequently implicated in a broad spectrum of respiratory disorders ranging from mild allergy to severe, life-threatening invasive diseases (1, 2). Among the more prevalent conditions associated with *A. fumigatus* are steroid-unresponsive bronchial asthma, allergic bronchopulmonary aspergillosis (ABPA), aspergilloma, and invasive pulmonary aspergillosis (IPA), particularly in immunocompromised individuals such as those undergoing chemotherapy or organ transplantation. The global burden of *Aspergillus*-related respiratory diseases is well documented (3). Because of the small size, *Aspergillus* conidia are capable of penetrating deep into the lower airways (4). Once inside the host, these spores release a variety of allergenic proteins and virulence factors that contribute to host sensitization and disease progression. The host immune response, especially in atopic individuals, frequently includes IgE-mediated sensitization to these proteins, which contributes to chronic airway inflammation and remodelling (5).

To date, over thirty allergens from *A. fumigatus* have been characterized and officially listed in the WHO/IUIS Allergen Nomenclature Database. Many of these allergens are extracellular in nature and include members of diverse protease families, such as alkaline serine proteases, metallo-proteases, and aspartic proteases. These enzymes are not only immunogenic but also play critical roles in the fungus's pathogenic strategy by degrading host tissue components, facilitating fungal invasion, and modulating immune responses. Among them, aspartic proteases, commonly referred to as aspergillopepsins, are of particular interest due to their dual role as allergens and virulence factors (6–8).

One such allergen is Asp f 10, an aspartic endopeptidase identified in AF conidia and reported to be secreted during active fungal invasion of host tissues (7). Despite its registration in the WHO/IUIS allergen database, Asp f 10 remains one of the least characterized allergens in terms of biochemical structure, immunological properties, and clinical diagnostic potential. A previous serological study reported that recombinant Asp f 10 showed IgE-reactivity in approximately 28% of ABPA patients, suggesting its potential role in host sensitization. However, the allergen's structural properties, enzymatic activity, and differential seroreactivity in various clinical contexts remain largely unexplored (9).

In allergic diseases, elevated levels of allergen-specific IgE-antibodies are widely recognized as indicators of immediate-type hypersensitivity. Consequently, measurement of specific IgE is a standard approach in allergy diagnosis. However, IgG antibodies, particularly in chronic fungal exposure, represent another arm of the adaptive immune response, reflecting antigen exposure history and immune recognition without necessarily leading to allergic inflammation. In the context of ABPA and other Aspergillus-associated conditions, IgG levels can serve as important diagnostic indicators, especially when IgE results are equivocal or when chronic colonization complicates clinical interpretation (10).

In the present study, we focused our analysis on IgE and total IgG, rather than IgM or IgG subclasses, for both biological and practical reasons. IgM, being the first immunoglobulin isotype produced after antigen exposure, typically indicates acute infection rather than chronic or allergic responses and is therefore not routinely assessed in fungal allergy diagnostics. While IgG subclasses (IgG1 to IgG4) may offer additional resolution in understanding immune polarization, subclass-specific assays are not standardized for most fungal antigens and are seldom used in clinical diagnostics due to cost, variability, and lack of validated thresholds (11). Total IgG, in contrast, provides a reliable and scalable approach in detecting antigen-specific humoral immunity and has been previously validated in serodiagnosis of fungal infections, including chronic pulmonary aspergillosis and ABPA (12).

Here, we undertook a comprehensive biochemical and serological characterization of Asp f 10. Our goal was to determine the diagnostic potential of recombinant Asp f 10 particularly in the context of IgG and IgE responses and to assess whether it can serve as a reliable serological biomarker for ABPA and other AF-associated respiratory diseases.

## Materials and methods

*Human Subjects:* This study was approved by the Institutional Human Ethics Committee (Approval No. PU-HEC-A/IHS/2023/001/2 dated May 19, 2023). Written informed consent was obtained from all participants. Blood samples were collected anonymized. Patient demographics are summarized in Table 1. Six different cohorts were recruited in the present study such as ABPA, SAAS (severs asthma with *Asperigllus* sensitization), HDM (house dust mite allergy), FA (food allergy), UTI (urinary tract infection), and non-atopic healthy (NA).

**Table 1:** Clinical and demographic details of human subject cohorts.

Patient cohort	Abbreviation	Number	Mean age ± SD (Years)	Sex	Total IgE Mean ± SD (kU/L)	Selection criteria
Allergic Bronchopulmonary Aspergillosis	ABPA	*n* = 50	41 ± 8	M = 28	441.8 ± 64.6	Total IgE > 500 IU/mL, positive Aspergillus fumigatus-specific IgG, and central bronchiectasis and high-attenuation mucus (22)
				F = 22		
Severe Asthma with Aspergillus Sensitization	SAAS	*n* = 41	39 ± 12	M = 29	311.4 ± 55.2	Total IgE < 500 IU/mL, negative Aspergillus fumigatus-specific IgG, no central bronchiectasis and high-attenuation mucus (22)
				F = 12		
House Dust Mite allergy	HDM	*n* = 84	44 ± 7	M = 37	242.1 ± 83.6	Elevated total IgE, specific IgE against HDM extract, positive skin reaction to HDM extract, and indoor allergy symptoms, no specific IgE against Aspergillus and food allergens (24)
				F = 47		
Food Allergy	FA	*n* = 52	Not known	Not known	321.4 ± 73.8	Typical food allergy symptoms and high titters of specific IgE to common food allergen extracts (25)
Urinary Tract Infection	UTI	*n* = 25	Not known	M = 3	38.6 ± 8.3	Normal level of total IgE, no history of any allergy, recent history of UTI (mostly with as diagnosed by elevated C-reactive protein, urine pus cells more than 8/hpf and C/S test (E. coli and Klebsiella) (26)
				F = 22		
Respiratory Tract Infection	RTI	*n* = 25	37 ± 12	M = 14	29.6 ± 7.6	Normal level of total IgE, no history of any allergy, no Aspergillus-specific IgG but recent history of community-acquired respiratory infection as evident from physical examination, neutrophil count and radiological assessment
				F = 11		

Demographic details were not available for the FA and UTI cohorts, as these groups were included solely as comparator cohorts to represent non-Aspergillus allergic and non-allergic baselines, respectively, rather than for primary inferential analysis.

*Extracellular Proteome Profiling:* AF strain (Accession No. IL4371) was procured from the National Culture Collection of Pathogenic Fungi (NCCPF), Chandigarh, in March 2021. Cultures were grown in minimal media supplemented with filter-sterilized goat lung extract (1:10 v/v) at 25 °C for 15 days. The culture supernatant was filtered and centrifuged to obtain the extracellular fraction, which was precipitated using 20% acetone overnight at 4 °C. The resulting protein pellet was washed with acetone containing 5 mM DTT and resuspended in rehydration buffer with 1% ampholytes (BioRad). Isoelectric focusing was conducted on Protean i12 IEF system (BioRad) using IPG strips pH 4–7 (BioRad), followed by second-dimensional SDS-PAGE (12%) and Coomassie staining.

*Immunoblotting:* Proteins from the 2D gel were transferred to PVDF membranes. After blocking with 5% BSA, membranes were incubated with pooled sera from four ABPA patients (1:100 v/v). IgG-reactive spots were detected using anti-human IgG-AP conjugate (1:1,000 v/v) and visualized with NBT/BCIP.

*Mass Spectrometry:* IgG-reactive protein spots were excised, trypsin digested and subjected to nano-LC-MS/MS for identification by C-CAMP, Bangalore, India.

*Cloning and recombinant expression:* A codon-optimized full-length Asp f 10 gene (1,188 bp) was synthesized (GenScript). The mature Asp f 10 fragment (975 bp) was PCR-amplified using the full-length construct as template. Both constructs were cloned into the pHisTEV expression vector using BamHI and XhoI restriction sites, resulting in recombinant proteins carrying an N-terminal 6 × His tag. Recombinant protein expression was induced in E. coli BL21(DE3) Rosetta cells with 0.5 mM IPTG at 16 °C for 12 h. Cells were harvested by centrifugation, resuspended in lysis buffer (1:50 w/v), and disrupted by sonication. Full-length Asp f 10 (fAsp f 10) was expressed predominantly as inclusion bodies and was therefore purified under denaturing conditions. The cell pellet was solubilized in denaturing lysis buffer containing 7 M urea, 250 mM Tris-HCl (pH 8.0), 300 mM NaCl, and 10 mM imidazole. The clarified lysate was incubated overnight at 4 °C with Ni-NTA resin (Qiagen). Unbound proteins were removed by washing with the same buffer supplemented with 40 mM imidazole. Bound proteins were eluted using buffer containing 250 mM imidazole under denaturing conditions. Purified fAsp f 10 (1 mg/mL) was subsequently solubilized by 20-fold rapid dilution into redox buffer (20 mM Tris-HCl, pH 8.0, 0.5 mM reduced glutathione, 0.005 mM oxidized glutathione, 0.05 mM glycine, 0.5 mM DTT, and 5% glycerol). The solubilized protein was concentrated to approximately 1.2 mg/mL using an Amicon Ultra centrifugal filtration unit (Merck Millipore). In contrast, mature Asp f 10 (mAsp f 10) was expressed in soluble form and purified under native conditions using Ni-NTA resin with the same buffer composition as described above, but without urea.

*Immunodot Blot:* About 100 ng of purified proteins were dotted onto nitrocellulose membranes, blocked with 5% BSA, and probed with individual patient sera (IgG: 1:100 v/v, IgE: 1:10 v/v). Detection was performed using monoclonal anti-human IgG or IgE-AP conjugates at 1:1,000 v/v (Sigma aldrich) and NBT/BCIP.

*Urea Unfolding Assay:* Soluble proteins (1 µg) were incubated with increasing concentrations of urea (0–8 M). Intrinsic tryptophan fluorescence was measured at excitation 280 nm and emission 337/350 nm. Protein stability was analysed based on the I_337_/I_350_ ratio as a function of urea concentration as described in (13).

*Size Exclusion Chromatography:* Approximately 0.5 mg of purified mAsp f 10 was loaded onto an Enrich SEC 70 size-exclusion chromatography column (Bio-Rad) pre-equilibrated with 25 mM Tris–HCl buffer containing 300 mM NaCl (pH 8.0) and connected to a BioLogic DuoFlow FPLC system (Bio-Rad). Prior to sample injection, the column was calibrated using a standard protein molecular weight mixture under identical buffer conditions. Protein elution was monitored by measuring absorbance at 280 nm, and fractions corresponding to the major elution peak were collected for further analysis.

*Circular Dichroism Spectroscopy:* Far-UV circular dichroism (CD) spectra of mAsp f 10 (∼0.8 mg/mL) were recorded at 25 °C using a J-1,500 CD spectrophotometer (JASCO Inc., Japan) over a wavelength range of 190–260 nm at a scanning speed of 1 nm s⁻¹. Spectra were acquired as the average of three consecutive scans and automatically corrected by subtracting the corresponding buffer baseline. The resulting spectra were analysed for secondary structure content using the CAPITO server.

*Protease Assays:* Bovine serum albumin (BSA; 1 mg/mL) was prepared in 50 mM sodium acetate buffer (pH 3.5) and used as the substrate for protease activity assays. Approximately 5 µg of either mature Asp f 10 (mAsp f 10) or full-length Asp f 10 (fAsp f 10) was added to the reaction mixture and incubated at 37 °C for 45 min. Commercial porcine pepsin (1 µg; SRL, India) served as a positive control. For inhibition studies, the enzyme was pre-incubated with 1 µg of pepstatin A (Sigma-Aldrich) prior to substrate addition. Final volume of the reaction mix was 500 μL. Reactions were terminated by adding trichloroacetic acid (TCA) to a final concentration of 10% (w/v) and incubated on ice to precipitate undigested proteins. Enzyme blank reactions, containing all components except protease, were included for each experimental set. Following centrifugation, the absorbance of TCA-soluble peptides in the supernatant was measured at 280 nm. An increase in absorbance was interpreted as an indicator of proteolytic activity. In addition, gelatin zymography was performed to qualitatively assess protease activity. Briefly, 2 µg of either porcine pepsin or Asp f 10 (with or without Pepstatin A) was resolved by native PAGE containing 0.02% (w/v) gelatin. Gels were subsequently incubated overnight at 37 °C in 0.1 M Tris–glycine buffer (pH 7.5). Proteolytic activity was visualized as clear lytic zones following Coomassie Brilliant Blue R-250 staining.

*Indirect ELISA:* ELISA plates (Maxisorp, Nunc) were coated with fAsp f 10 or mAsp f 10 (10 ng/µL in 50 μL reaction volume) and blocked with 3% BSA. Sera were added (IgG: 1:100 v/v, IgE: 1:10 v/v) and incubated at 4 °C for 10 h. Bound antibodies were detected with monoclonal anti-human IgG or IgE-AP conjugates at 1:1,000 v/v (Sigma Aldrich) both at 1:1,000 v/v followed by addition of pNPP substrate. Reactions were stopped with 3N NaOH and absorbance was read at 405 nm.

**Figure 1:**
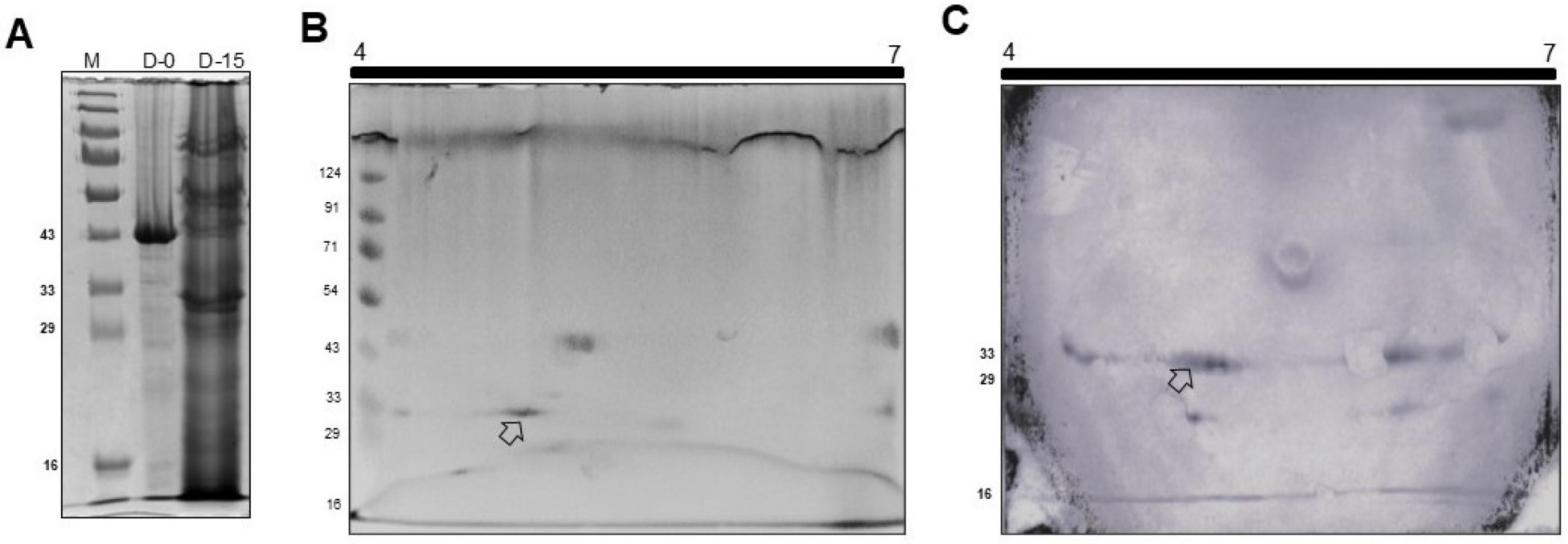
Serological analysis of the Aspergillus fumigatus (AF) secretome. **(A)** A. fumigatus was cultured for 15 days in minimal medium supplemented with goat lung extract. Culture supernatants were collected by centrifugation on day 0 (D-0, prior to inoculation) and day 15 (D-15, post-culture). Proteins were resolved by SDS-PAGE. **(B)** Two-dimensional gel electrophoresis of the extracellular (secreted) proteome of AF using immobilized pH gradient (IPG) strips spanning pH 4-7 in the first dimension, followed by 12.5% SDS-PAGE in the second dimension. Proteins were visualized by Coomassie Brilliant Blue R-250 (CBB-R250) staining. **(C)** Immunoblot of the AF secretome using pooled sera from four patients. IgG-reactive spots were detected, indicating immunoreactive extracellular proteins.

*Sample inclusion strategies:* In this study, the RTI cohort was recruited as a clinical negative control to determine whether bacterial infection, in the absence of allergy or *Aspergillus* sensitization, could produce non-specific Asp f 10 seroreactivity. As RTI antibody profiles overlapped with the non-allergic UTI baseline, these samples were excluded from inter-group modelling to preserve biologically meaningful contrasts, but are retained in Table 1 to demonstrate that acute respiratory infection does not confound Asp f 10 responses. The term “control” is used in two analytical contexts: the UTI cohort served as the global non-allergic reference for antibody thresholding and visualization baselines, whereas for intra-group case–control comparisons (Figures 1A,B; Supplementary Table S1), “control” denotes the non-case subset within each clinical category. Inter-group modelling analyses were therefore performed using case samples only, with the UTI mean shown solely as a visual baseline.

*Data pre-processing and quality control:* Data were imported into R (version 4.4.2) and screened for missing values, which were handled via list wise deletion. For inter-group analyses using case samples from each clinical cohort, RTI was excluded to prioritize allergic and fungal-related clinical cohorts, resulting in five groups: ABPA, SAAS, HDM, FA, and UTI. Antibody levels were log10-transformed (with a small offset of 0.001 to avoid undefined logs) to normalize distributions, followed by z-scoring for standardized comparisons. Binary flags were created for ABPA vs. non-ABPA classification. Analyses utilized packages including tidyverse, rstatix, pROC, MASS, ggpubr, dplyr, tidyr, and FSA.

*Threshold Determination for Antibody Positivity*: Positivity thresholds were established using the UTI group as a reference for non-allergic baseline reactivity. Thresholds were computed as the mean +2 standard deviations (SD) of raw IgE (0.063) and IgG (0.258) values in UTI participants, assuming approximate normality and capturing the 95% upper confidence limit. These cutoffs were used to classify positivity but were not directly plotted; instead, log10-transformed UTI means served as baselines in visualizations for interpretability.

*Statistical Analyses:* All statistical analyses were conducted in R (v4.4.2) In this study, classification of “case” and “non-case” samples within each clinical cohort was based solely on established clinical diagnostic criteria and was independent of Asp f 10-specific antibody levels. Within each cohort, “case” refers to individuals fulfilling diagnostic criteria for the respective condition, whereas “non-case” refers to individuals evaluated within the same clinical setting who did not meet those criteria. These non-case subsets represent clinically defined comparators and are not stratified based on antigen-specific immune responses. Antibody measurements (IgE and IgG) were log10-transformed to reduce skewness and stabilize variance. Normality assessment using Q–Q plots is provided in Supplementary Figure S4. For descriptive summaries, we report means, case–control differences, and fold changes. Intra-group comparisons between case vs. non-case samples within clinical cohorts were performed using two-sample *t*-tests on log10 values, with results expressed as both *p*-values and fold-change estimates (10^*Δ*log). For inter-group analyses restricted to cases (excluding RTI), global differences were tested using the Kruskal–Wallis rank-sum test. *post hoc* pairwise comparisons were conducted with Dunn's tests, applying the Benjamini–Hochberg (BH) procedure to control the false discovery rate. Effect sizes were quantified using epsilon-squared [*ε*² = (*χ*²−*k* + 1)/(*n*−*k*)], where *χ*^2^ is the test statistic, *k* the number of groups, and *n* the total sample size. To evaluate diagnostic performance for identifying ABPA against all non-ABPA groups, receiver operating characteristic (ROC) curves were constructed for z-scored IgE and IgG values using the *pROC* package. Area under the curve (AUC) values with 95% confidence intervals were computed via bootstrap resampling. For classification analysis, separate linear discriminant analysis (LDA) models were fitted for IgE and IgG (MASS package). Predictors were z-scored antibody levels, and group membership served as the categorical response. Model performance was quantified using leave-one-out cross-validation (LOOCV), with both overall accuracy and balanced accuracy (mean recall across groups) reported. Note that for ROC and LDA analyses, the “non-ABPA” group was defined as case samples from SAAS, HDM, FA, and UTI cohorts. To visualize separation, individual projections onto the first linear discriminant axis (LD1) were displayed as group-wise boxplots.

*Histamine Release Assay***:** mAsp f 10 was incubated with 200 µL whole blood from four ABPA patients known to be IgE-seropositive for Asp f 3. Histamine release was quantified using a commercial EIA kit (Cayman Chemical) and percentage of release was calculated as described in (14). A > 5% release was considered positive.

## Results

**Figure 2:**
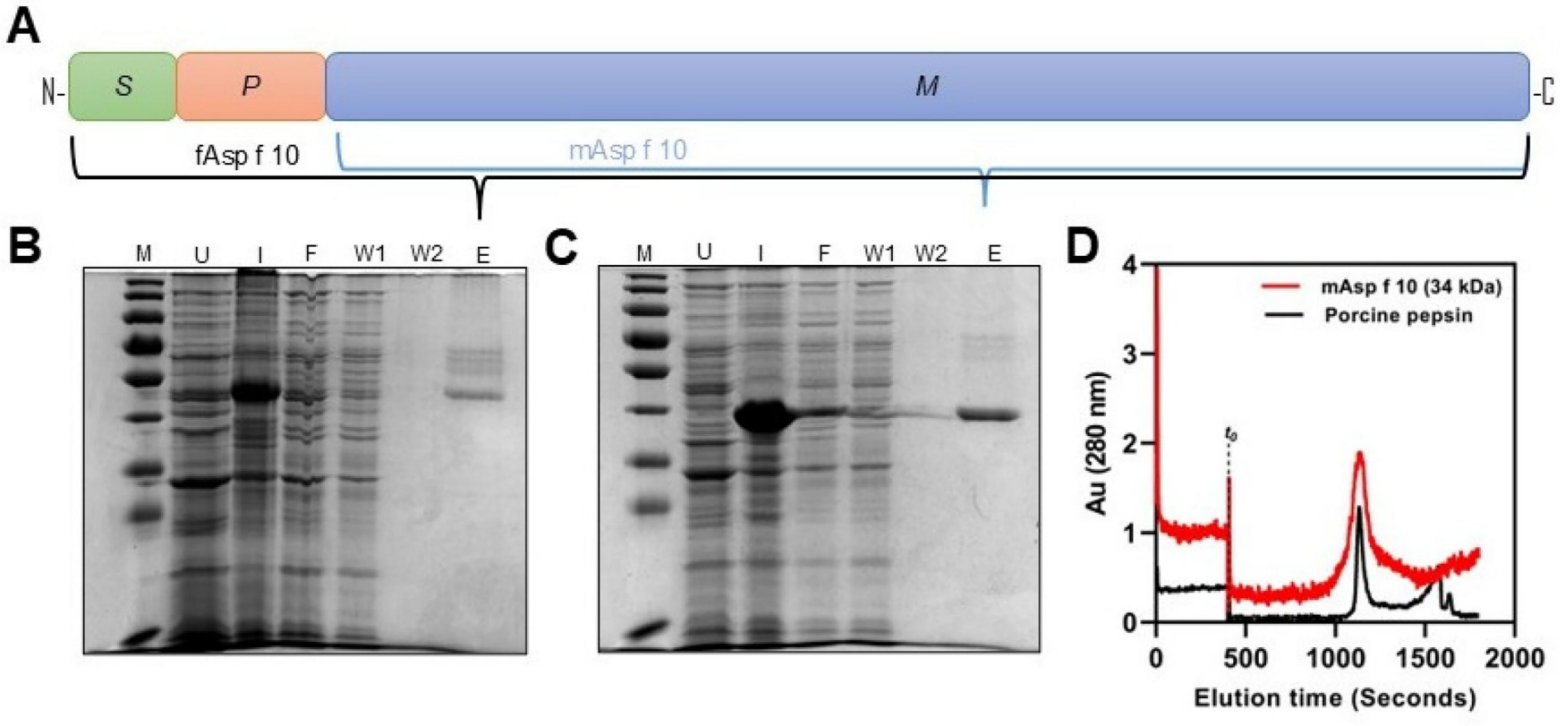
Purification and immunochemical analysis of recombinant Asp f 10. **(A)** Schematic representation of the domain architecture of full-length Asp f 10 (fAsp f 10), comprising an N-terminal 29-residue signal peptide (M1-G29, S), a 40-residue propeptide (F30-A70, P), and the 325-residue mature enzyme (S71-A395, M). Both fAsp f 10 and the mature form (mAsp f 10) were cloned into a bacterial expression vector and expressed as recombinant proteins in E. coli. **(B,C)** SDS-PAGE profiles showing stepwise purification of 6 × His-tagged fAsp f 10 and mAsp f 10 using metal affinity chromatography. Lane U: uninduced E. coli lysate; Lane I: lysate after IPTG induction; Lane F: flow-through post Ni-NTA binding; Lanes W1 and W2: wash fractions (40 mM imidazole); Lane E: elution with 250 mM imidazole. **(D)** Size-exclusion chromatography elution profile of mAsp f 10. The protein eluted at the same retention volume as porcine pepsin (35 kDa), indicating similar molecular weight.

*AF secretome contains an IgG-reactive Aspartic Protease:* Minimal media supplemented with goat lung extract effectively mimicked pulmonary conditions. After 15 days, several proteins were secreted by AF as shown in Figure 1A. Immunoblotting of AF secretome shown in Figures 1B,C revealed a 34 kDa, pI 4.2 protein reacting with patient IgG. LC-MS/MS identified it as Aspergillopepsin, with 32% sequence coverage and 5 unique peptides. Sequence analysis matched 100% with the known allergen Asp f 10. Full-length Asp f 10 consists of a 29-residue signal peptide, a 40-residue propeptide, and a 324-residue mature domain (Figure 2A).

**Figure 3:**
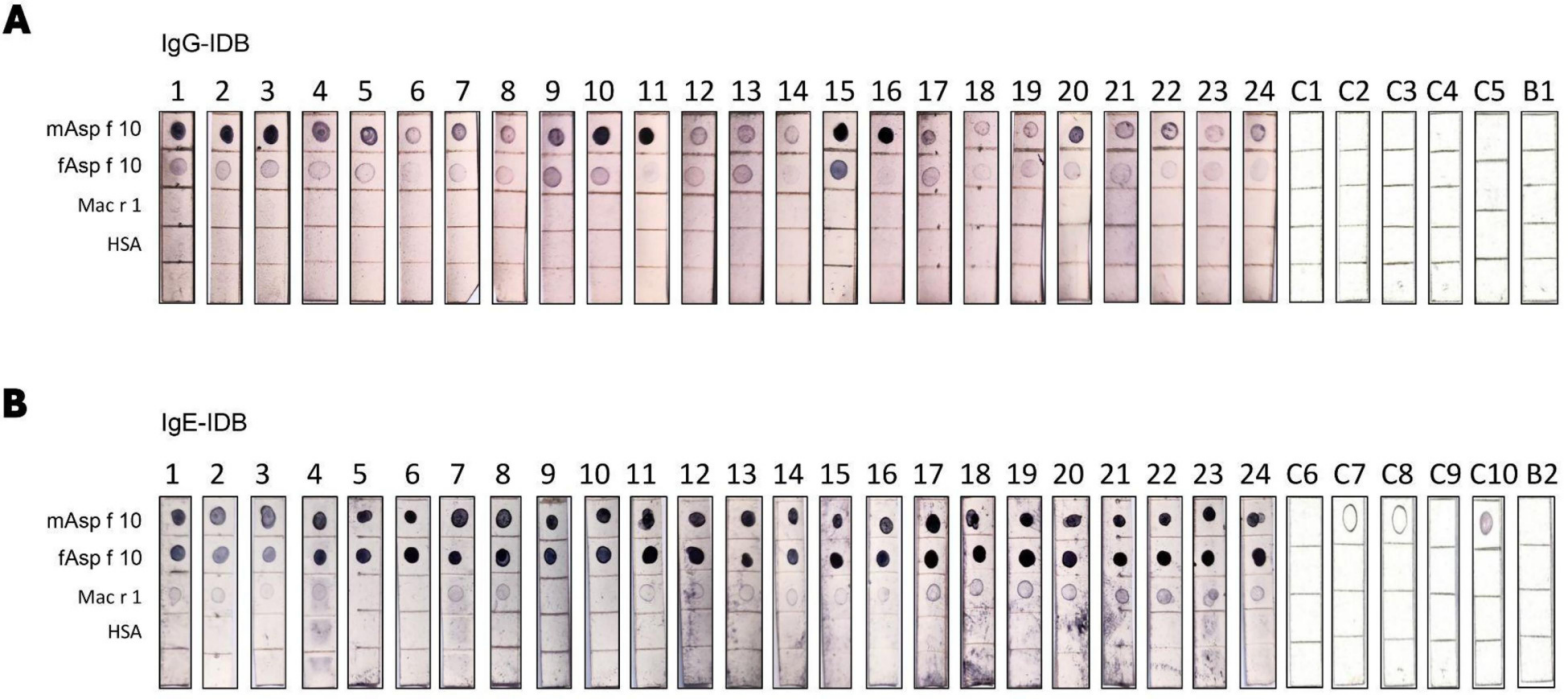
Immunochemical analysis of recombinant Asp f 10 **(A)** IgG- and IgE-immunodot blot analysis of 100 ng each of fAsp f 10 and mAsp f 10 using sera from ABPA patients (*n* = 12; strips 1-12) and SAAS patients (*n* = 12; strips 13-24). Equal amounts of Mac r 1 (prawn allergen) and human serum albumin (HSA) were used as negative control antigens. Sera from non-atopic individuals were used as negative controls (strips C1-C5 for IgG and strips C6-C10 for IgE immunoblots). Strips B1 and B2 represent buffer controls.

*Recombinant Asp f 10 shows seroreactivity:* fAsp f 10 and mAsp f 10 were expressed in *E. coli* as inclusion bodies and soluble form respectively with a final yield of 3–4 mg per Litre of culture (Figures 2B,C). In size exclusion column mAsp f 10 appeared as a single peak at an elution time very close to porcine stomach pepsin of molecular weight 34 kDa (Figure 2D). Refolded fAsp f 10 failed to elute as a defined peak. Qualitative immuno-dot blot analysis in Figures 3A,B revealed the presence of IgG and IgE-reactivity in both fAsp f 10 as well as mAsp f 10 when tested with ABPA positive patient sera. Although the IgG-reactive dots of fAsp f 10 appeared to be much faint as compared to mAsp f 10. Antigens used as negative controls neither reacted with IgG nor with IgE.

**Figure 4:**
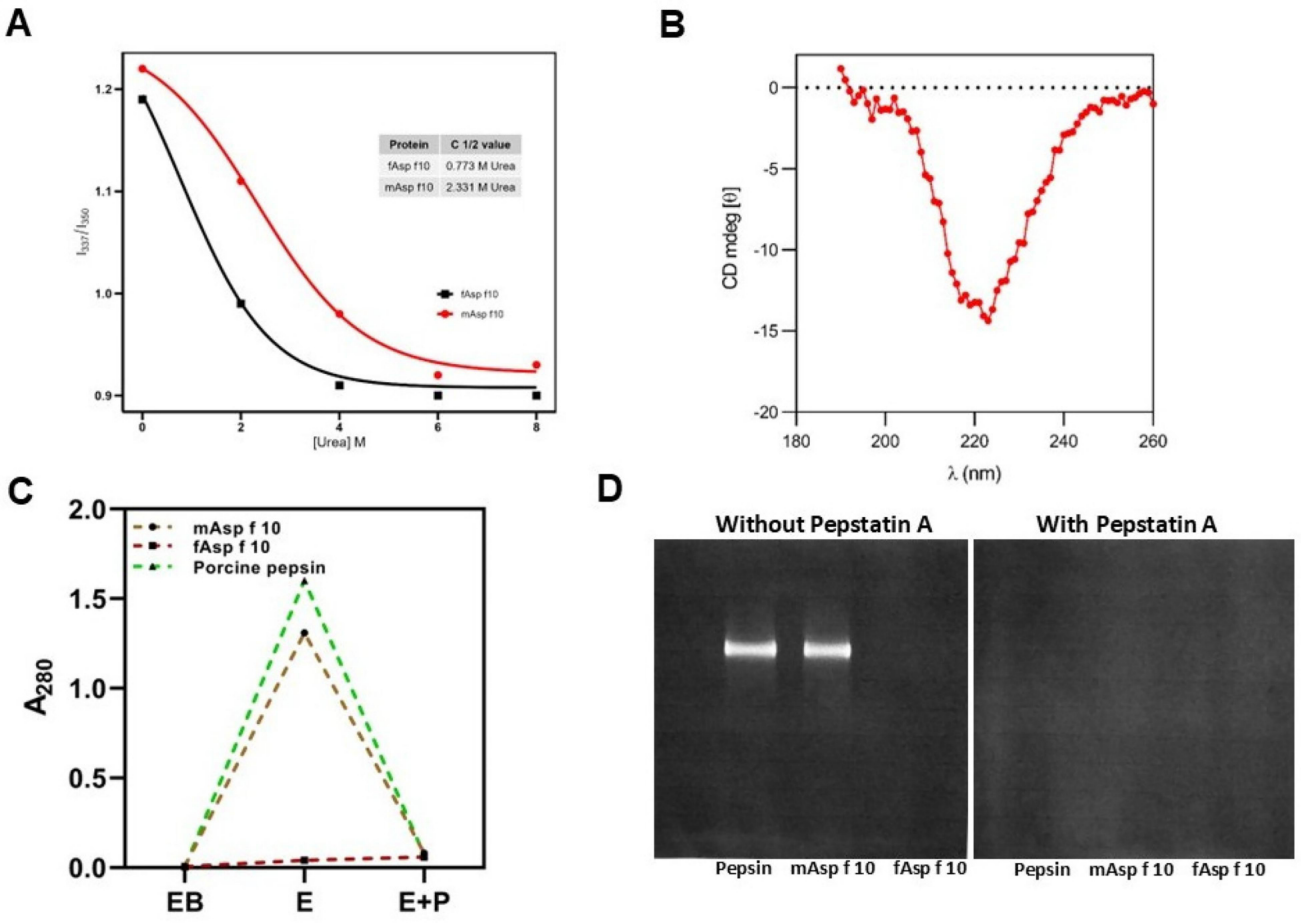
Functional characterization of Asp f 10. (**A)** Equilibrium urea-induced unfolding profiles of full-length Asp f 10 (fAsp f 10) and mature Asp f 10 (mAsp f 10) at 25 °C. Proteins were incubated for 16 h in increasing concentrations of urea prepared in 0.05 M Tris buffer containing 100 mM NaCl (pH 8.0), followed by measurement of intrinsic tryptophan fluorescence. The ratio of emission intensities at 337 nm and 350 nm (excitation at 295 nm) was plotted against urea concentration. Symbols represent experimental data points; solid lines indicate the best fit to a three-state unfolding model. The inset shows the urea concentration at which 50% denaturation (C½) was achieved for each protein. **(C)** Protease activity of mAsp f 10 and fAsp f 10 was assessed spectrophotometrically by incubating the enzymes with bovine serum albumin (BSA) at enzyme-to-substrate ratios of 1:100 for Asp f 10 and 1:500 for porcine pepsin. Reaction progress was monitored by measuring the absorbance of TCA-soluble peptides at 280 nm. A sharp increase in A280 was observed in the enzyme reaction **(E)** compared with the enzyme blank (EB). Pre-incubation of mAsp f 10 with pepstatin A (E + P) inhibited enzymatic activity, confirming its identity as an aspartic protease. Commercially available porcine pepsin was used as a positive control. **(D)** Enzymatic activity of mAsp f 10 was assessed by gelatin zymography, showing a clear lytic zone indicative of proteolytic activity. In contrast, fAsp f 10 showed no detectable activity. Protease activity was completely inhibited upon pre-incubation with pepstatin A.

*mAsp f 10 is structurally stable and functionally active:* Urea denaturation showed a sigmoidal transition with C1/2 of 2.3 M for mAsp f 10, vs. 0.773 M for fAsp f 10, suggesting increased stability in the mature form (Figure 4A). CD analysis confirmed a properly folded protein structure with 39.6% antiparallel beta sheet for mAsp f 10 (Figure 4B), while fAsp f 10 lacked defined secondary structure (data not shown). The enzyme assay shown in Figure 4C revealed significant protease activity in mAsp f 10, as evident from a sharp increase in the A_280_ of TCA-soluble peptides generated from BSA digestion compared with the enzyme blank control. A similar activity pattern was observed for commercially available pepsin used as a positive control. In contrast, fAsp f 10 lacked detectable enzymatic activity. Pre-incubation of mAsp f 10 with pepstatin A resulted in a marked inhibition of proteolytic activity, confirming Asp f 10 as an aspartic protease. Zymography confirmed gelatinolytic activity of mAsp f 10 (Figure 4D) but no activity was observed for full-length protein.

**Figure 5:**
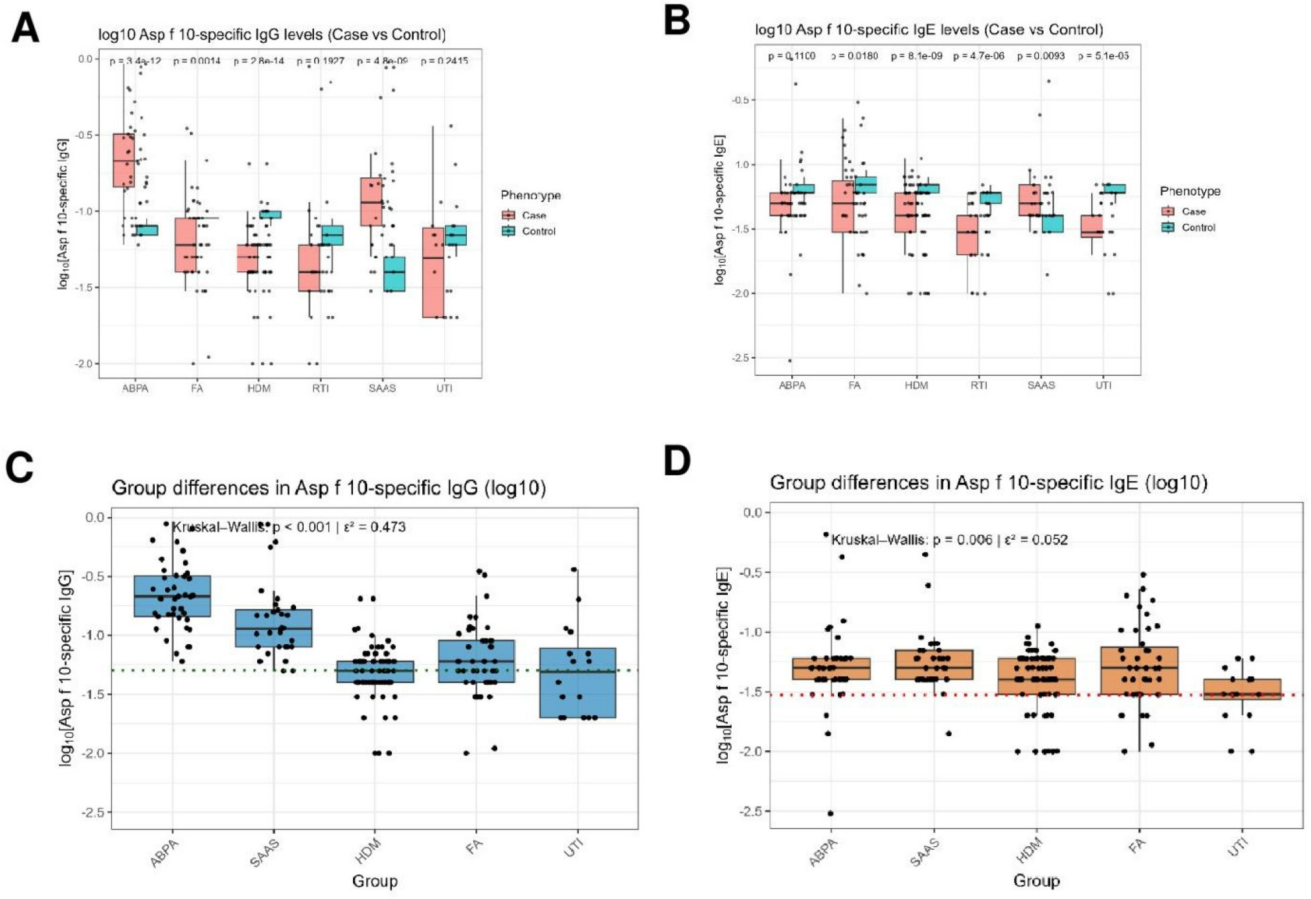
Serological reactivity to Asp f 10 in clinical cohorts. **(A,B)** Boxplots of log₁₀-transformed (z-scored) Asp f 10-specific antibody levels showing intra-cohort comparisons between case and non-case samples within each clinical cohort (ABPA, *n* = 50; SAAS, *n* = 41; HDM, *n* = 84; FA, *n* = 52). The term “control” refers to non-case samples within the same cohort and does not represent the UTI group. Log₁₀ transformation was applied to reduce skewness inherent to ELISA measurements. The UTI cohort (*n* = 25) is shown separately as a non-allergic baseline reference. Two-sided t-tests were used for intra-cohort comparisons (IgG: ABPA *p* = 3.36 × 10⁻^12^, fold-change = 2.87; SAAS *p* = 4.80 × 10⁻^9^, fold-change = 3.33; IgE: non-significant for ABPA, *p* = 0.110, fold-change = 0.80). **(C,D)** Inter-group comparisons among allergic cases (ABPA, SAAS, HDM, FA, UTI) using Kruskal–Wallis tests followed by Dunn's *post-hoc* tests with Benjamini–Hochberg correction (IgG: *χ*² = 99.5, d.f. = 4, *p* = 1.26 × 10⁻²⁰, *ε*² = 0.473; ABPA vs. UTI Z = 2.98, p_adj = 0.014; IgE: *χ*² = 14.4, d.f. = 4, *p* = 0.006, *ε*² = 0.052; ABPA vs. UTI Z = 2.979, p_adj = 0.014). Dotted lines indicate UTI baselines. Data points represent individual samples; boxes span interquartile ranges with medians as central lines; whiskers extend to 1.5× interquartile ranges. ns, not significant; *p_adj < 0.05, **p_adj < 0.01, ***p_adj < 10⁻³. **(A,B)** Intra-cohort comparisons of log10-transformed Asp f 10-specific antibody levels between case and non-case samples within each clinical cohort (ABPA, SAAS, HDM, FA). The term “control” refers to non-case samples within the same cohort and does not represent the UTI group. Log10 transformation was applied to reduce skewness inherent to ELISA-based measurements. In this figure, case and non-case classifications are based on clinical criteria and are independent of Asp f 10-specific antibody levels.

*Asp f 10-specific IgG shows pronounced elevations in ABPA and SAAS cases relative to controls:* Intergroup comparisons revealed significant differences in Asp f 10-specific antibody levels between Cases and Controls. For IgG, t-tests indicated highly significant elevations in case samples of ABPA (*p* = 3.36 × 10⁻¹²), and SAAS (*p* = 4.80 × 10⁻⁹); and control samples of HDM (*p* = 2.81 × 10⁻¹⁴), and FA (*p* = 0.001), with non-significant changes in RTI (*p* = 0.193) and UTI (*p* = 0.242). Fold-change analysis quantified these elevations, showing 2.87-fold higher IgG in ABPA case vs. non-case samples within clinical cohorts and 3.33-fold in SAAS, while other groups exhibited downregulation (e.g., 0.53-fold in HDM). Mean log10 differences underscored this pattern, with positive shifts in ABPA (0.458) and SAAS (0.523), contrasting with negative differences elsewhere. IgE responses were less consistent, with significant elevations in FA (*p* = 0.018), HDM (*p* = 8.06 × 10⁻⁹), RTI (*p* = 4.69 × 10⁻⁶), SAAS (*p* = 0.009), and UTI (*p* = 5.06 × 10⁻⁵), but not ABPA (*p* = 0.110). Fold changes for IgE were generally modest, with SAAS showing a 1.44-fold increase and ABPA a 0.80-fold decrease, (Supplementary Table S1) highlighting IgE's limited specificity for ABPA. In non-*Aspergillus* cohorts (HDM, FA, RTI, UTI), fold changes below unity reflect the absence of Asp f 10-specific immune enrichment in clinically defined case groups, consistent with cohort classification independent of fungal antigen reactivity. Boxplots (Figures 5A,B) visually confirmed these trends, with statistical annotations emphasizing IgG's stronger case-control distinctions.

*Inter-group differences highlight IgG's discriminative power among cases:* Focusing on cases across ABPA, SAAS, HDM, FA, and UTI, Kruskal–Wallis tests demonstrated significant global differences for both the antibody classes, but with markedly higher effect sizes for IgG (*χ*² = 99.5, df = 4, *p* = 1.26 × 10⁻^20^, *ε*² = 0.473) than IgE (*χ*² = 14.4, df = 4, *p* = 0.006, *ε*² = 0.052). Pairwise Dunn's tests with BH adjustment for IgG revealed highly significant elevations in ABPA and SAAS vs. others (Supplementary Figure S2). For IgE, significance limited to UTI vs. all groups, but other pairs remain non-significant (Supplementary Figure S3). Boxplots (Figures 5C,D) illustrated these inter-group variations on the log10 scale, with dotted lines indicating UTI baselines. IgG levels in ABPA and SAAS Cases clustered above baselines, reinforcing their fungal-sensitized profiles, while IgE showed broader overlap.

**Figure 6:**
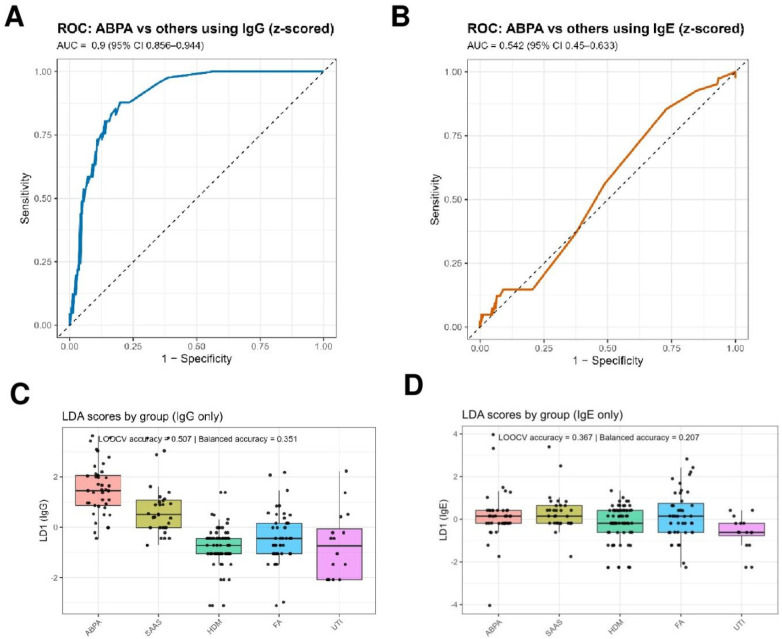
Diagnostic performance of Asp f 10-specific antibodies for ABPA classification. **(A,B)** Receiver operating characteristic (ROC) curves distinguishing ABPA from non-ABPA cases using z-scored IgG (AUC = 0.900, 95% CI 0.856–0.944) or IgE (AUC = 0.542, 95% CI 0.450–0.633) levels, computed via bootstrap resampling (*n* = 1,000). **(C,D)** Linear discriminant analysis (LDA) projections onto the first discriminant axis (LD1) for group separation among cases (IgG: leave-one-out cross-validation accuracy = 0.507, balanced accuracy = 0.351; IgE: accuracy = 0.367, balanced accuracy = 0.207). Data points represent individual samples; boxes span interquartile ranges with medians as central lines; whiskers extend to 1.5 ×  interquartile ranges. Colors denote groups as in Figure 5.

*ROC Analysis Affirms IgG's Diagnostic Superiority for ABPA:* ROC curves for distinguishing ABPA from non-ABPA cases (using z-scored markers) yielded an AUC of 0.900 for IgG (95% CI: 0.856–0.944), indicating excellent discriminative capacity. This outperformed IgE (AUC = 0.542, 95% CI: 0.450–0.633), which hovered near random chance. Curves (Figures 6A,B) depicted clear separation for IgG, supporting its utility in clinical settings where ABPA must be differentiated from atopic conditions like HDM or SAAS.

*LDA Demonstrates Enhanced Group Separation with IgG:* Separate LDA models confirmed IgG's dominance in multivariate space. For IgG, LOOCV accuracy was 0.507 with balanced accuracy of 0.351, reflecting moderate overall classification but strong ABPA isolation along LD1. IgE performed poorly (LOOCV = 0.367, balanced = 0.207), with substantial group overlap. Projections (Figures 6C,D) showed ABPA distinctly separated via IgG, while SAAS exhibited partial distinction, underscoring IgG's role in resolving diagnostic overlaps.

**Figure 7:**
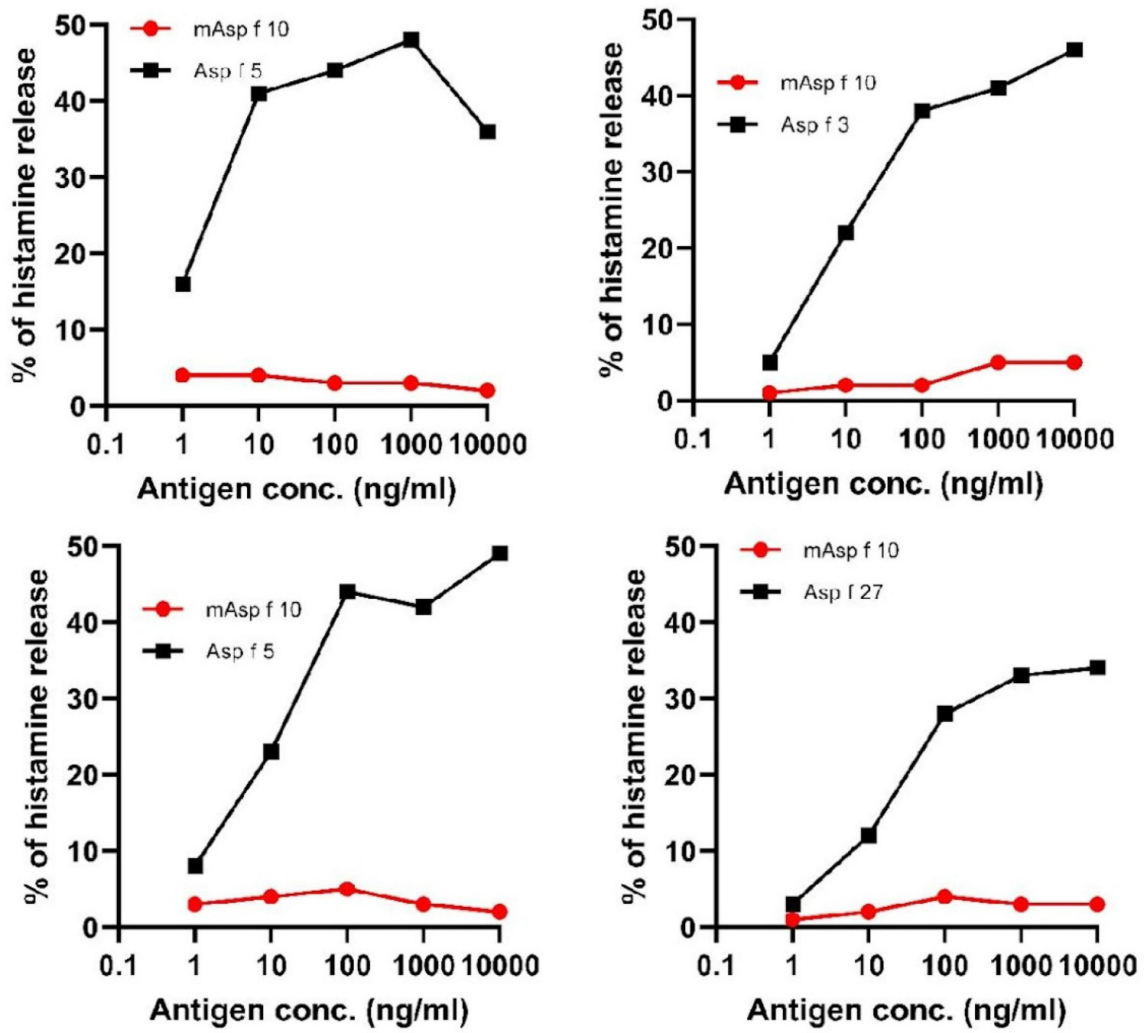
Histamine release assay in *Aspergillus*-sensitized patients. Peripheral blood from four Aspergillus-sensitized patients was stimulated with increasing concentrations of mAsp f 10. The percentage of histamine released was plotted against allergen concentration. A threshold of 5% histamine release was set, above which responses were considered indicative of positive basophil degranulation. As a positive control, blood samples were stimulated with a second allergen to which each patient had a confirmed high specific IgE titre.

*mAsp f 10 Fails to Induce Histamine Release:* Despite high IgE-reactivity, mAsp f 10 failed to induce histamine release from basophils in any of the four tested ABPA patients. In contrast, control allergens triggered significant degranulation, with EC50 values < 100 ng/mL shown in Figure 7.

## Discussion

This study describes a comprehensive characterization of Asp f 10, a secreted aspartic protease from *Aspergillus fumigatus*, and highlights its utility as a diagnostic marker for ABPA. Although previously reported as a minor allergen, Asp f 10 has remained poorly characterized in terms of antigenicity, and diagnostic relevance (15–17). The present study fills this knowledge gap and underscore the discriminatory power of Asp f 10-specific IgG responses in differentiating ABPA from other *Aspergillus*-associated and non-fungal allergic conditions.

Presence of Asp f 10 as a major extracellular IgG-reactive protein in AF secretome confirms its expression and immunogenicity during ABPA. Recombinant expression of Asp f 10 allowed further biochemical and immunological investigation. Notably, the matured form i.e., mAsp f 10 folded into a stable, β-sheet-rich conformation. Functionally, mAsp f 10 displayed enzymatic properties consistent with other fungal aspartyl proteases (18). This suggests that Asp f 10 is secreted in an active, structurally resilient form that may contribute to pathogenesis. However, further studies are warranted to elucidate its precise role in degrading tissue barriers and host invasion (19).

Antibody binding analyses revealed that both fAsp f 10 and mAsp f 10 were recognized by sera from ABPA patients. However, fAsp f 10 exhibited lower IgG signal intensity than mAsp f 10, likely due to conformational epitope differences following proteolytic maturation (20). Importantly, qualitative immuno-dot blot assays demonstrated the presence of IgE reactivity against mAsp f 10, whereas quantitative ELISA revealed that the overall IgE-binding titres were low. This apparent discrepancy can be attributed not only to the qualitative vs. quantitative nature of the assays but also to differences in antigen presentation. In dot blot, proteins are immobilized at high local concentrations with heterogeneous distribution on the membrane, which can facilitate detection of low-affinity or low-abundance IgE interactions. In contrast, ELISA involves adsorption of antigen as a relatively uniform monolayer, where epitope accessibility, orientation, and density are more constrained, thereby providing a more stringent assessment of antibody binding. Furthermore, functional evaluation through histamine release assay demonstrated that mAsp f 10 failed to induce basophil degranulation even in patients exhibiting comparatively higher IgE titres. Together, these findings indicate that detectable IgE binding in an immunoblot does not necessarily translate into functional allergenicity, as effective effector cell activation requires sufficient epitope density and spatial arrangement for Fc*ε*RI cross-linking. Such observations are consistent with previous reports describing IgE-reactive proteins that lack effector cell activation capacity (21, 22). The data therefore suggest that Asp f 10 may represent an IgE-binding but functionally weak allergen, possibly influenced by suboptimal folding or limited presentation of conformational epitopes, reinforcing the importance of combining qualitative, quantitative, and functional assays for accurate allergen characterization.

Among all serological parameters evaluated, Asp f 10-specific IgG responses emerged as the most informative marker for ABPA. Statistically significant elevations in IgG levels were observed in ABPA patients compared to SAAS, HDM, FA, and UTI groups. Importantly, IgG levels were able to discriminate ABPA from SAAS, a clinically overlapping yet pathologically distinct condition. Notably, SAAS patients classified as negative for A*. fumigatus*–specific IgG by commercial extract-based assays exhibited elevated Asp f 10–specific IgG responses. This discrepancy highlights a limitation of crude extract diagnostics, which may underrepresent antibodies to secreted virulence-associated allergens. Antigen-resolved serology thus reveals Aspergillus-directed immune recognition not captured by current tests and suggests that a subset of SAAS patients with Asp f 10-IgG positivity may lie closer to the ABPA end of the disease spectrum. Additionally, the high area under the ROC curve (AUC = 0.9) and clear separation along the LDA axis reinforce the diagnostic robustness of Asp f 10-specific IgG. On contrary, Asp f 10-specific IgE responses, though detectable, showed modest intergroup differences and limited specificity, rendering them less useful in differential diagnosis.

These findings also establish the importance of IgG-based diagnostics in fungal allergy. Traditionally, IgE has been used as the principal marker in allergic disease. However, its specificity and sensitivity often decrease in complex conditions like ABPA, where immune responses are influenced by persistent fungal colonization. The use of high-specificity IgG, particularly against virulence-associated fungal antigens such as Asp f 10, represents a novel paradigm in allergy diagnostics and may complement the existing IgE-based assays or serve as an independent tool when IgE results are equivocal (23).

Our findings indicate that Asp f 10–specific IgG holds strong diagnostic potential, but they should be interpreted within the context of the evolving consensus on ABPA criteria. The revised ISHAM-ABPA guidelines (22) currently classify Aspergillus fumigatus specific IgG as one of three ancillary criteria, along with blood eosinophilia and radiologic findings, of which any two may suffice for diagnosis. Our study demonstrates that quantitative Asp f 10–IgG not only meets but surpasses the diagnostic performance of total or specific IgE, suggesting that more refined IgG-based assays could meaningfully strengthen existing diagnostic frameworks. However, given that current ISHAM recommendations are founded on multi-cohort international validation, any proposal to elevate IgG to an obligatory criterion must be supported by larger, multicentric datasets and longitudinal studies assessing reproducibility, clinical outcomes, and treatment response. A key limitation of this study is the absence of detailed clinical outcome data for the Asp f 10-IgG-positive SAAS subgroup. Parameters such as lung function, exacerbation frequency, and corticosteroid requirement were not available, precluding assessment of whether this immunologically defined subset differs in clinically meaningful ways. Additionally, detailed demographic and clinical characterization of case and non-case subgroups within cohorts was not performed, as the study was designed to evaluate antigen-specific serological responses rather than matched intra-cohort comparisons. Future studies integrating antigen-resolved serology with comprehensive clinical phenotyping will be essential to establish the clinical relevance of these findings. In summary, this study positions Asp f 10-specific IgG as a promising serological biomarker with high diagnostic value for ABPA. The ability to distinguish ABPA from SAAS and other non-fungal allergic cohorts using a single fungal protein offers a major step forward in allergy diagnostics. Moreover, the biochemical features of mAsp f 10 support its biological relevance and potential for inclusion in allergen panels or microarray platforms. Future studies should evaluate its performance in larger cohorts and explore its inclusion in multiplex assays for comprehensive fungal allergy profiling.

## Data Availability

The original contributions presented in the study are included in the article/Supplementary Material, further inquiries can be directed to the corresponding authors.
