# Supplementary material for: Serological and biochemical characterization of Aspergillus fumigatus Asp f 10 as a potential diagnostic marker in ABPA and related respiratory diseases
Source: Front Allergy. 2026 Jun 5;7:1733299. doi: 10.3389/falgy.2026.1733299 (PMC13279211; doi:10.3389/falgy.2026.1733299)
Supplement: Supplementary file 1 [file Table1.docx]

Table S1: Positivity summary of case versus control

Table S2: Results of Dunn’s test for IgG

Table S3: Results of Dunn’s test for IgE
