# Supplementary material for: Serological and biochemical characterization of Aspergillus fumigatus Asp f 10 as a potential diagnostic marker in ABPA and related respiratory diseases
Source: Front Allergy. 2026 Jun 5;7:1733299. doi: 10.3389/falgy.2026.1733299 (PMC13279211; doi:10.3389/falgy.2026.1733299)

Table S1: Positivity summary of case versus control. Geometric means were recalculated directly from raw ELISA values using log-transformation and back-transformation.

Table S2: Results of Dunn’s test for IgG

Table S3: Results of Dunn’s test for IgE

Figure S4: Q–Q plots showing distribution of log10-transformed Asp f 10-specific IgE (A) and IgG (B) values across clinical cohorts. The transformation substantially improved symmetry of distributions, supporting the use of parametric scaling for visualization, while statistical analyses were conducted using non-parametric methods.

**A)**


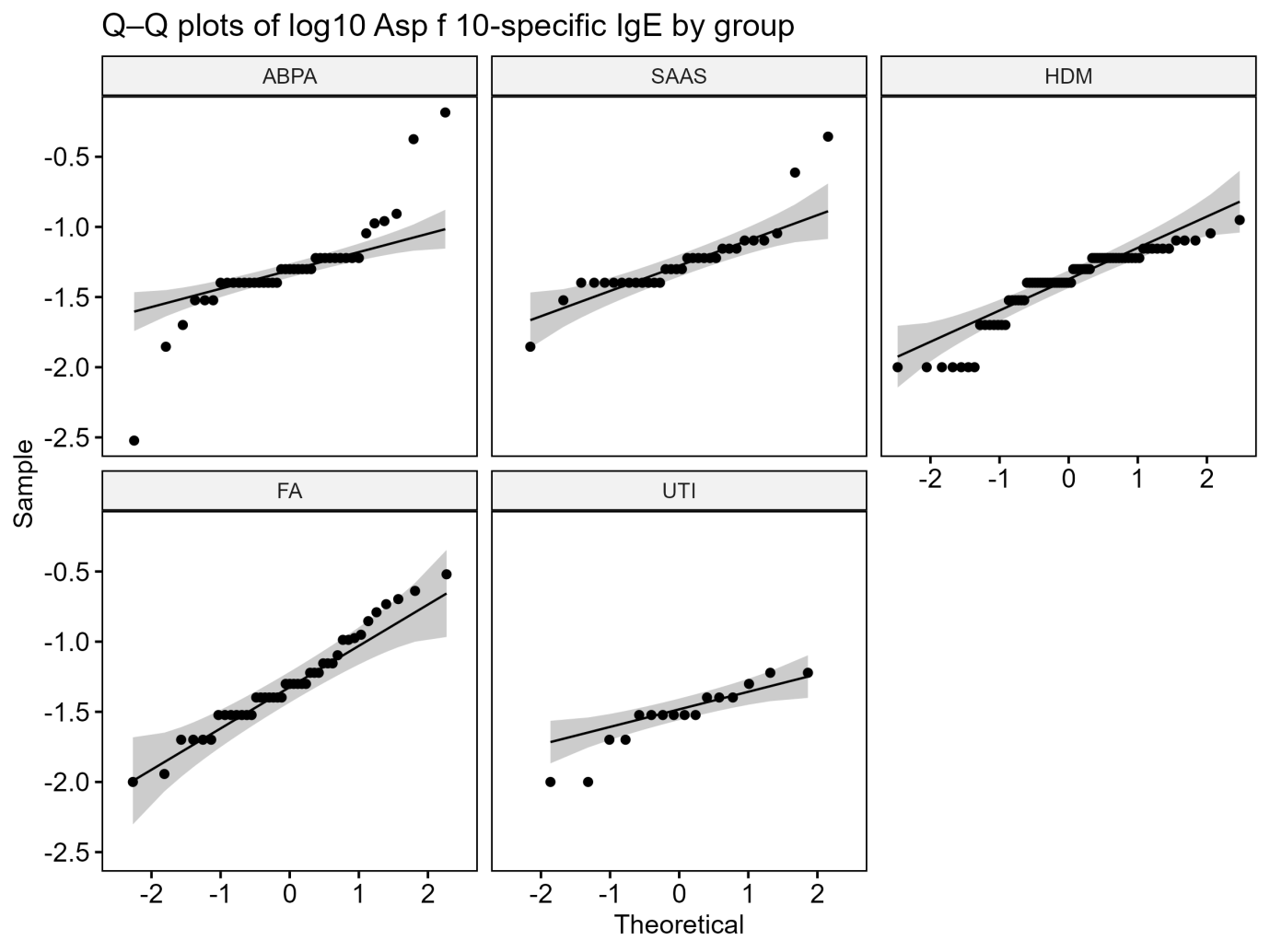


**B)**


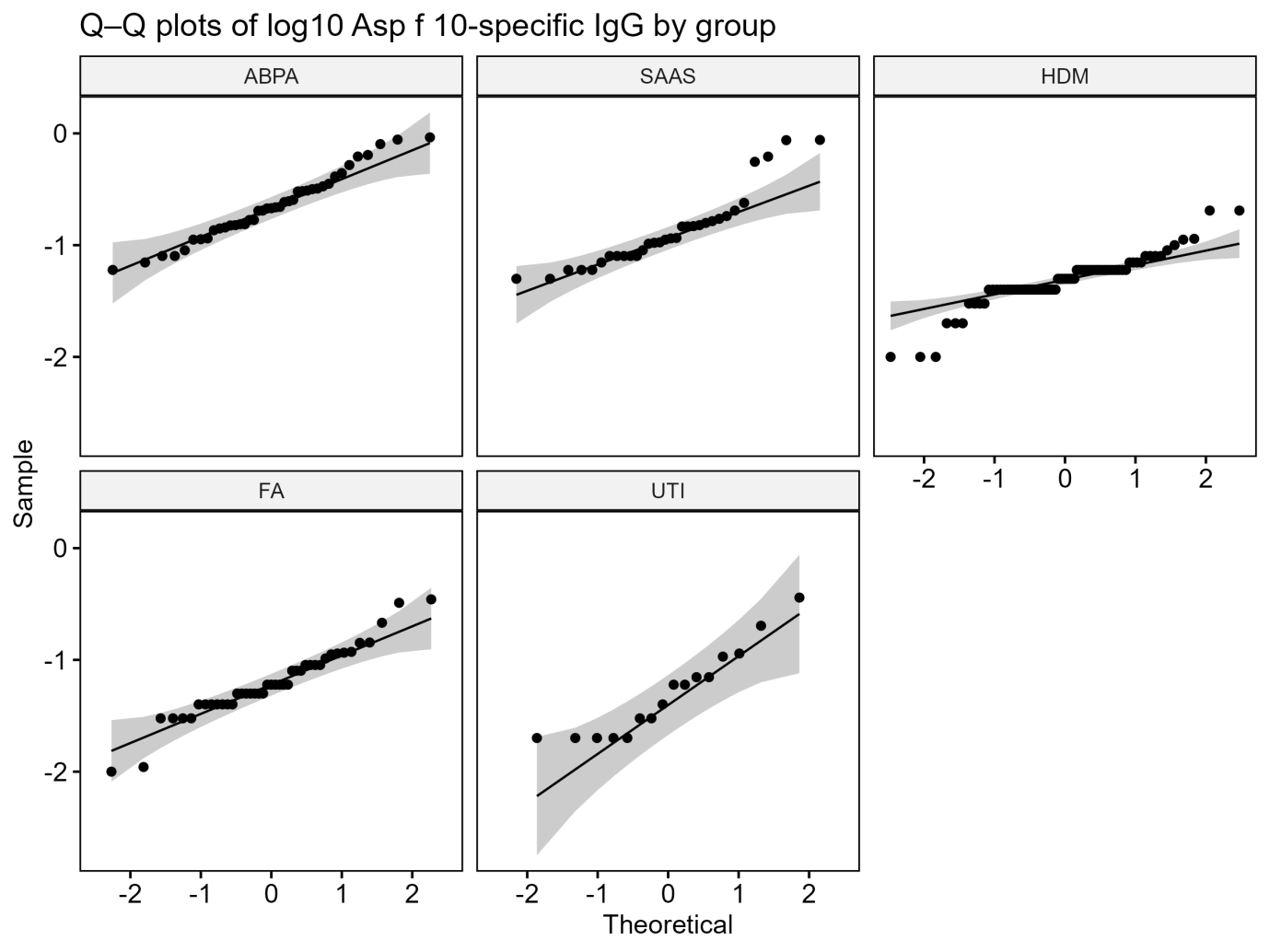

Supplement: Supplementary file 2 [file Table5.docx]
